# Curettage and Artificial Bone Graft Using Chevron Osteotomy for Bone Tumor of the Talus: A Report of Three Cases

**DOI:** 10.7759/cureus.92490

**Published:** 2025-09-16

**Authors:** Yoshiro Yoshikawa, Hiromichi Oshiro, Yuta Miyashi, Yasunori Tome, Kotaro Nishida

**Affiliations:** 1 Department of Orthopedic Surgery, Graduate School of Medicine, University of the Ryukyus, Ginowan, JPN

**Keywords:** aneurysmal bone cyst, benign bone tumor, chevron-type medial malleolar osteotomy, giant cell tumor of the bone, talar bone tumor, talus

## Abstract

Primary talar bone tumors are rare. Surgical intervention on talar bone tumors is often challenging, even though most lesions are benign. Due to its unique anatomical and biomechanical characteristics, this surgical approach requires careful consideration. Although most benign talar tumors can be treated with curettage alone, insufficient visualization increases the risk of recurrence, making the selection of an appropriate surgical approach crucial. We performed a chevron-type medial malleolar osteotomy, a technique widely used for its excellent exposure and stability, to manage talar tumors. Herein, we report three cases of benign talar tumors that were successfully treated with chevron-type medial malleolar osteotomy. Case 1, with a giant cell tumor of the bone (GCTB), experienced recurrence after initial curettage but was managed effectively with improved exposure using chevron-type medial malleolar osteotomy. Using the chevron-type osteotomy, case 2 and 3 patients with aneurysmal bone cysts (ABC) and GCTB, respectively, had no recurrence or complications. The medial malleolus was osteotomized in a reverse V-shape while preserving the deltoid ligament and was flipped distally for exposure. This technique offers excellent visualization and stability, suggesting its utility in managing talar tumors. This three-case series is the first to report the use of a chevron-type medial malleolar osteotomy for managing talar bone tumors, potentially aiding the surgical management of talar tumors, particularly those involving the talar dome.

## Introduction

Primary talar bone tumors are uncommon [1]. Although most lesions are benign, surgical intervention is required to maintain the talar function, such as weight-bearing and ankle motion. Because of its unique anatomical and biomechanical characteristics, the surgical approach must be carefully considered to ensure appropriate tumor management [2]. Various techniques for medial malleolar osteotomy have been developed [3-5]. However, performing surgery on talar bone tumors is challenging due to the complex three-dimensional anatomical structure of the ankle joint [6]. Here, we report our experience with chevron-type medial malleolar osteotomy for benign bone tumors of the talus, which provided adequate intraoperative exposure and resulted in no recurrences or complications [7]. The method and schematic are presented below. Guided by Kirschner wires (K-wires), a reverse V-shaped osteotomy of the medial malleolus was performed, preserving the deltoid ligament, and the fragment was hinged distally to expose the talus.

## Case presentation

Surgical procedures are as follows: the patient was placed in a supine position under general anesthesia with the use of a tourniquet. An approximately 6 cm longitudinal skin incision was made on the medial malleolus. After the exposure of the medial malleolus, a Kirschner wire (K-wire) was inserted from the distal tibia to the medial corner of the ankle mortise using an X-ray image intensifier. A reverse V-shaped osteotomy of the medial malleolus was performed along the guide K-wire, and the osteomized fragment preserving the deltoid ligament was hinged distally to expose the medial talar wall. A 3 × 3 cm-sized cortical window was created in the medial talar wall, after intralesional curettage with a high-speed burr and phenol and ethanol ablations as adjuvant treatments if needed. The defect was filled with α-tricalcium phosphate (α-TCP) (Biopex®, Tokyo, Japan). Then, the cortical bone lid was returned and adhered to α-TCP, restoring the cortical contour. The medial malleolus was returned and fixed with two canulated screws.

Case 1

**Figure 1:**
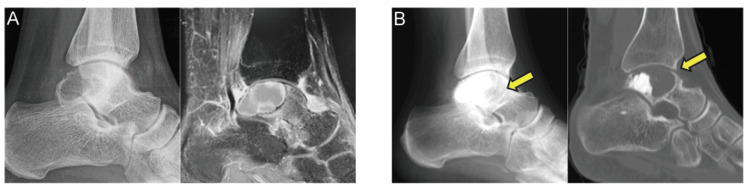
Case 1: initial images. (A) (Left) Radiograph shows an osteolytic change in the posterior talus. (Right) MRI demonstrates a talar bone tumor exhibiting rim enhancement on a gadolinium-enhanced T1 fat-suppressed image (right). (B) Findings of local recurrence six months after the initial surgery: (left) postoperative radiograph reveals osteolytic lesions surrounding the artificial bone graft (arrows) and newly developed osteoarthritic changes that were absent preoperatively. (Right) CT image shows talar dome collapse with osteoarthritic changes in the tibiotalar joint (arrows). MRI, magnetic resonance imaging; CT, computed tomography

A 22-year-old man presented with a four-month atraumatic left ankle pain exacerbated by dorsiflexion and weight-bearing. The radiography and computed tomography (CT) imaging of the left ankle revealed talar osteolysis and a lesion with low signal on T1-weighted image/high signal on T2-weighted image, fluid-fluid levels, and peripheral gadolinium enhancement on magnetic resonance imaging (MRI), suggesting a giant cell tumor of the bone (GCTB) or an aneurysmal bone cyst (ABC) (Figure 1A). Radiographic evaluations revealed no evidence of osteoarthritic changes. The initial surgery was performed via a posterolateral approach, with a 7 cm longitudinal incision made in the posterior aspect of the distal fibula, which enabled the cortical fenestration of the talus and the curettage of the hematoma-like material. Rapid pathology indicated ABC; nonetheless, the final pathological examination confirmed GCTB. The cavity was filled with α-tricalcium phosphate (α-TCP) (Biopex®, Tokyo, Japan), and the cortical flap was replaced. Six months postoperatively, radiographs and CT images revealed radiolucent areas consistent with tumor recurrence while also exhibiting osteoarthritic changes in the tibiotalar joint (Figure 1B).

**Figure 2:**
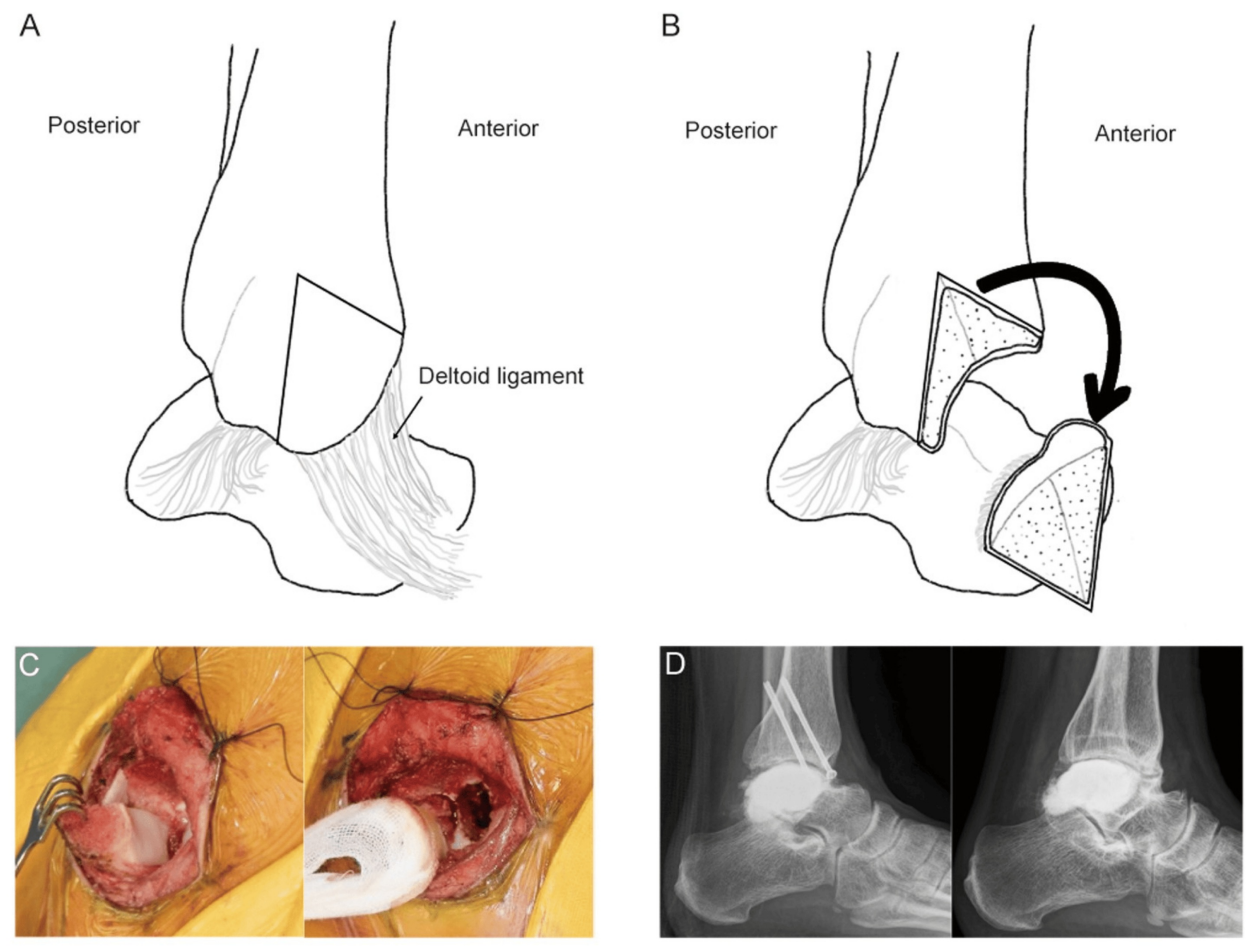
Case 1: schemas illustrating the chevron-type medial malleolar osteotomy, intraoperative photographs, and images. (A) After the apex is identified, a reverse-V chevron osteotomy is performed. (B) With the deltoid ligament left intact, the osteotomized fragment is retracted distally, thereby exposing the talus. (C) Intraoperative view: (left) the image confirms that a chevron‑type medial malleolar osteotomy with an intact deltoid ligament permits adequate talar exposure. (Right) The image demonstrates curettage, followed by phenol/ethanol ablation under clear visualization. (D) Postoperative radiographs: the left image was obtained immediately after re-surgery, whereas the Right image, taken at the latest follow-up 20 months post re-surgery, shows no evidence of recurrence despite residual osteoarthritic change and the collapse of the medial talar dome. Image credits: Yoshiro Yoshikawa.

A second surgery was performed one year following the initial surgery, employing a chevron-type medial malleolar osteotomy. After creating the osteotomy trajectory with a K-wire, the medial malleolus was osteotomized in a reverse V-shaped manner while preserving the deltoid ligament continuity and then flipped distally to expose the talus (Figure 2A, 2B, 2C). A cortical window was created in the medial aspect of the talar wall, and curettage was performed using a high-speed burr. After the curettage of the recurrent tumor, three cycles of phenol and ethanol ablation, and irrigation with distilled water, the defect was filled with α-TCP. The medial malleolus was then returned to its original position and fixed using two cannulated screws. The patient remained non-weight-bearing for four weeks postoperatively, followed by gradual weight-bearing. At the three-year follow-up, the osteotomized medial malleolus had achieved bone union without evidence of talar necrosis or recurrence (Figure 2D). Osteoarthritic changes in the tibiotalar joint persisted following initial surgery, and the patient complained of mild left ankle pain; nevertheless, he could return to work at the last follow-up.

Case 2

**Figure 3:**
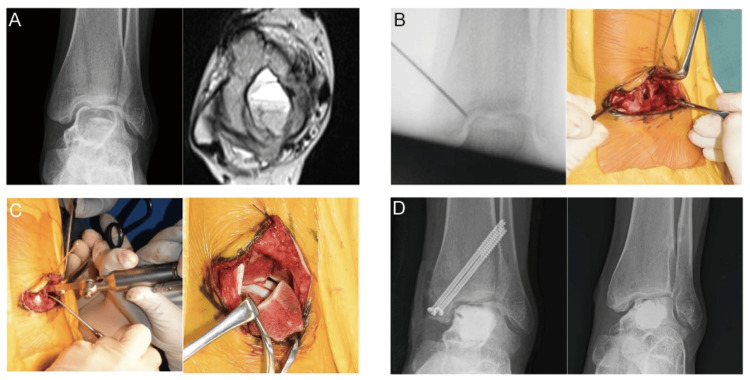
Case 2: pre- and postoperative images and intraoperative photographs. (A) (Left) Radiograph demonstrates an osteolytic lesion in the talar body, and preoperative osteoarthritic change is already evident. (Right) MRI depicts a bone tumor with a fluid-fluid level, respectively. (B) Intraoperative findings: the left image fluoroscopically confirms the apex of the reverse‑V chevron osteotomy, whereas the right image shows a U-shaped skin incision with dissection extended through the subcutaneous and fascial layers. (C) Osteotomy and talar exposure: the left image depicts the performance of the chevron-type medial malleolar osteotomy, whereas the right image shows the preservation of the deltoid ligament and the distal reflection of the osteotomized fragment for talar exposure. (D) Postoperative radiographs: the left image was obtained immediately after surgery, whereas the right image, taken at the 39-month follow-up, demonstrates no evidence of recurrence despite residual osteoarthritic change and the collapse of the medial talar dome. MRI: magnetic resonance imaging

A 13-year-old girl presented with left ankle pain with no apparent cause. Examinations revealed tenderness over the medial ankle in dorsiflexion and plantar flexion. Radiography and CT imaging showed osteolytic changes and the collapse of the left talar dome. MRI demonstrated a lesion with low signal on T1-weighted images and high signal on T2-weighted images, fluid-fluid levels, and gadolinium rim enhancement, suggesting GCTB or ABC (Figure 3A). Initially, a chevron-type medial malleolar osteotomy was performed. A skin incision was made along the medial malleolus, and part of the flexor digitorum longus tendon sheath was incised (Figure 3B). Subsequently, a reverse V-shaped osteotomy of the medial malleolus was carried out to expose the talus (Figure 3C). The cortical fenestration of the medial talar wall was then undertaken, and the hematoma-like material was curetted. Three sequential cycles of phenol and ethanol ablation were applied, followed by irrigation with distilled water. The defect was subsequently filled with α-TCP, and the osteotomized medial malleolus was fixed with two cannulated cancellous screws at the initial position. Postoperatively, the patient was immobilized and remained non-weight-bearing for four weeks, followed by a gradual return to weight-bearing. The final pathological examination confirmed an aneurysmal bone cyst. The cannulated screws were removed after one year. At the last follow-up, two years after surgery, the bone union of the osteotomized medial malleolus was confirmed, with no local recurrence (Figure 3D). At the last follow-up, the collapse of the talar dome persisted; however, the patient did not complain of ankle pain.

Case 3

**Figure 4:**
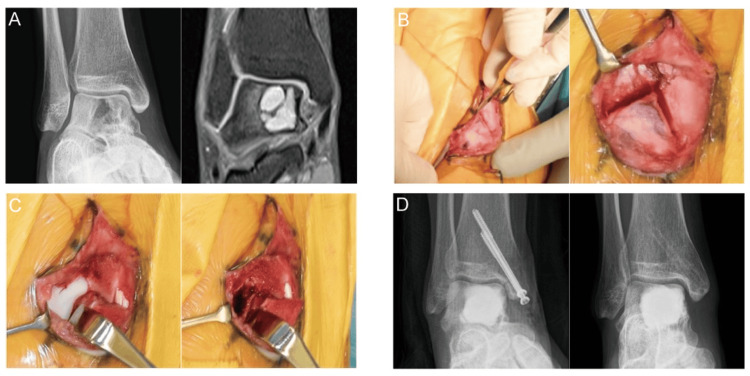
Case 3: pre- and postoperative images and intraoperative photographs. (A) (Left) Radiograph reveals an osteolytic change in the talar body, whereas (right) MRI displays a bone tumor with a lobulated internal architecture. (B) Intraoperative findings: the left* *image identifies the apex of the reverse-V chevron osteotomy, whereas the right image shows the execution of the chevron-type medial malleolar osteotomy. (C) After the distal reflection of the osteotomized fragment: the left image demonstrates the excellent exposure of the talar dome, whereas the right image depicts the creation of a cortical window, followed by curettage of the lesion. (D) Postoperative radiographs: the left image was obtained immediately after surgery, whereas the right image, taken at the 23-month follow-up, confirms solid union after implant removal with no evidence of recurrence or osteoarthritic degeneration. MRI: magnetic resonance imaging

A 25-year-old woman presented with atraumatic pain lasting six months in her right ankle. She reported that the pain had been worsening, especially in the front and rear portions, during dorsiflexion. Radiographs and CT imaging showed multiple osteolyses of the right talus, and MRI demonstrated lesions with low and high signal on T1-weighted and T2-weighted images, respectively, and gadolinium rim enhancement, suggesting a GCTB or an ABC (Figure 4A). A chevron-type medial malleolar osteotomy was performed (Figure 4B), followed by the curettage of the hematoma-like material and three cycles of phenol, ethanol, and distilled water (Figure 4C). The defect was filled with α-TCP, and the medial malleolus was fixed with two cannulated cancellous screws. Postoperatively, the patient was immobilized for four weeks and then gradually resumed weight-bearing. The final pathological examination confirmed GCTB. The cannulated screws were removed after one year. At the last follow-up, two years after surgery, the bone union of the osteotomized medial malleolus was achieved, with no local recurrence, talar necrosis, or osteoarthritic changes (Figure 4D).

## Discussion

Primary bone tumors of the talus are uncommon, and the available literature is scarce [1]. Foot bone tumors constitute approximately 3% of all skeletal tumors, with those affecting the talus accounting for 8%-23% of foot-related cases [8-10]. Bell et al. reviewed the Scottish Bone Tumour Registry, which comprises patients with primary bone tumors in Scotland, from January 1954 to May 2010, and identified 23 cases of tumors involving the talus [1]; additionally, 20 cases of benign tumors were found, including five, four, and one case of osteoid osteoma, chondroblastoma, and GCTB, respectively. Although most benign talar tumors can be managed with curettage alone, the choice of surgical exposure is critical because inadequate visualization may lead to incomplete curettage and subsequent recurrence.

In Case 1, we initially selected a posterior approach without medial malleolar osteotomy. Nonetheless, visualization was limited, and tumor access was restricted. Tumor recurrence was confirmed six months postoperatively. Therefore, we adopted an alternative approach.

To obtain better visualization, several medial malleolar osteotomy techniques have been developed, including transverse, oblique, step-cut, and U-shaped osteotomies, which, however, are associated with several intraoperative and postoperative complications [3-5]. Limited talus visualization, bone dislocation after surgery, nonunion after medial malleolar fixation, and technical difficulties have been previously reported [3-5].

Chevron osteotomy was first described in 1976 as an effective and safe treatment for hallux valgus [7]. Due to its high rotational stability and favorable bone healing rates, the technique was soon adapted for use at other sites. In 2002, Cohen and Anderson introduced a chevron-type medial malleolar osteotomy and reported excellent exposure in the talar dome [11]. Subsequent finite-element and cadaveric studies have confirmed its superior construct stability over oblique cuts, while clinical series consistently report union within six weeks in ≥95% of cases [12-14]. Currently, chevron osteotomy is widely used for medial malleolar access. Nonetheless, some reports have described chevron osteotomy in a talus tumorlike lesion (intraosseous ganglion cyst) or osteochondral lesion surgery [13,15]. This three-case series is the first to report chevron-type medial malleolar osteotomy for the management of talar bone tumors, to our knowledge. This approach provides a broad view of the talus, including the talar dome and body. This facilitates the intralesional management of bone tumors of the talus. However, the visualization of the talar neck or head is limited with this exposure, requiring an anterior or alternative approach.

Case 1 represented our first experience using a chevron-type medial malleolar osteotomy for a bone tumor. During the second operation in Case 1, this approach provided excellent exposure. Although osteoarthritic changes secondary to recurrence could not be prevented, no additional recurrence occurred. On the basis of this experience, we subsequently adopted chevron osteotomy as our primary treatment for talar tumors, particularly those involving the talar dome. In Cases 2 and 3, the same technique afforded adequate intraoperative exposure. While surgery does not reverse preexisting osteoarthritis, it achieves good local tumor control. Over two years of follow-up, we observed neither recurrence nor osteoarthritic changes attributable to the chevron osteotomy.

This study had some limitations. First, the sample size was small, and the follow-up was relatively short; therefore, a longer observation period is needed. Second, as our primary concern was recurrence, we did not collect patient-reported outcome measurements. Third, postoperative CT scans were not routinely obtained, which limited the evaluation of talar dome collapse. Future studies are warranted and should incorporate patient-reported outcome measures, such as the Foot and Ankle Outcome Score and the American Orthopaedic Foot and Ankle Society Score, as well as a standardized postoperative CT protocol to facilitate future discussions of the risk of complications and long-term outcomes.

## Conclusions

We performed a chevron-type medial malleolar osteotomy for benign talar bone tumors. Adequate intraoperative exposure was achieved, and no recurrence was observed in three cases. Preexisting tibiotalar joint osteoarthritis is irreversible; however, osteoarthritis progression was not observed. This technique ensures reliable local tumor control and favorable clinical outcomes.

Curettage and artificial bone graft using a chevron-type osteotomy for benign talar bone tumors may preserve the limb function of the ankle joint, although exposure and reconstruction are challenging due to the complex anatomy of the ankle joint.
